# GLIMMPSE Lite: Calculating Power and Sample Size on Smartphone Devices

**DOI:** 10.1371/journal.pone.0102082

**Published:** 2014-12-26

**Authors:** Aarti Munjal, Uttara R. Sakhadeo, Keith E. Muller, Deborah H. Glueck, Sarah M. Kreidler

**Affiliations:** 1 Dept. of Biostatistics and Informatics, University of Colorado Denver, Aurora, Colorado, United States of America; 2 Health Outcomes and Policy, University of Florida, Gainesville, Florida, United States of America; Shenzhen Institutes of Advanced Technology, China

## Abstract

Researchers seeking to develop complex statistical applications for mobile devices face a common set of difficult implementation issues. In this work, we discuss general solutions to the design challenges. We demonstrate the utility of the solutions for a free mobile application designed to provide power and sample size calculations for univariate, one-way analysis of variance (ANOVA), GLIMMPSE Lite. Our design decisions provide a guide for other scientists seeking to produce statistical software for mobile platforms.

## Introduction

### Motivation

An increasing number of scientists and statisticians use mobile devices, such as smart phones and tablets. Moving statistical power and sample size software to mobile devices allows designing studies anywhere, anytime, even when away from the office.

Developers of mobile statistical applications face a common set of design challenges. In this paper, we list the challenges and provide general solutions. In addition, we provide detailed example solutions from our experience developing GLIMMPSE Lite. GLIMMPSE Lite calculates power and sample size for studies that use analysis of variance (ANOVA). Our design decisions should be useful for other developers of statistical software aimed at mobile platforms.

### Migrating statistical power software to a mobile platform

We present a list of challenges we faced in designing statistical power software for a mobile platform. We encountered each one of the challenges listed below while designing our mobile power and sample size application, GLIMMPSE Lite.

#### C1. Statistical power software is computationally intensive, and many mobile devices have limited computing resources

Power calculations for ANOVA in particular, and the general linear model in general, involve complex matrix operations such as inversions and multiplication of large matrices. In addition, sample size calculations do not have a closed form, and require numerical search algorithms. Given that the calculations are both memory and processor intensive, statistical power applications may run poorly on mobile devices with limited random access memory (RAM) or slow processors.

#### C2. Statistical power software requires complex inputs

To perform a power or sample size calculation, a user must input several pieces of information about the design. Inputs include the Type I error rate, the list of independent and dependent variables, the hypothesis of interest, and choices for means and variances. Developing an intuitive user interface to accept these inputs is a challenge. For mobile devices, developers have an additional challenge of tailoring the interface to the small screen format.

#### C3. The interface must provide an appropriate level of guidance to users regarding the required inputs

Users of power and sample size software have differing levels of statistical training. Some users may need additional assistance to understand inputs to the power or sample size calculation. The limited screen size of a mobile device forces designers to present concise help pages that do not clutter the view.

#### C4. Communication between the mobile device and supporting servers must be fast and lightweight

Using a client-server architecture is a better option than performing power and sample size calculations directly on the mobile device. In the client-server architecture, the mobile device elicits inputs from the user and sends the data to a server with greater computing resources. In turn, the server performs the actual calculation. The client-server architecture solves the problem of limited computing resources on the mobile device, but introduces a potential bottleneck in communication with the server. Application performance may be optimal over wi-fi or 4G cellular networks, but performance may degrade on slower cellular data networks such as 2G and 3G. To maintain adequate performance at slower data speeds, the communication protocol must be both lightweight and optimally utilize available bandwidth.

#### C5. The mobile app must scale easily and robustly

With an increasing number of mobile device users and the potential for wide distribution from online application stores, the number of users for a popular application can increase very rapidly. Thus, the client-server architecture supporting the mobile application must scale well. This means that the application must handle an increasing number of users in a short time frame without failure.

#### C6. Supporting multiple platforms requires significant development time

Every mobile platform has its own software development kit (SDK). Applications developed for one mobile platform are not compatible with other mobile platforms. Thus, development time increases proportionally to the number of devices supported.

#### C7. Mobile devices have frequent updates in operating systems

Due to frequent updates, developers must ensure that any application is compatible with current versions of the mobile operating system and a reasonable subset of previous versions. The problem is compounded when supporting multiple brands of mobile devices.

#### C8. Applications must coordinate with other applications

Most mobile devices are capable of running multiple applications simultaneously. Therefore, a power and sample size application must manage shared resources such as file storage or network connections appropriately, and gracefully handle errors generated when interacting with other applications.

#### C9. Users must be able to save outputs for later reference

To increase the usability of power and sample size calculations on a mobile platform, users must be able to save outputs and use them later.

#### C10. Users must be able to reproduce the power and sample size results without reiterating the whole process

To facilitate reproducible research, scientists and statisticians need an accurate record of the steps involved in a power or data analysis. Therefore, users must be able to save any design, and recall it for reuse later.

### Mobile design by example: the GLIMMPSE Lite software

GLIMMPSE Lite is a smartphone application that calculates power and sample size for ANOVA study designs on two mobile platforms: iOS from Apple [1] and Android from Google [2]. GLIMMPSE Lite widens the user base of GLIMMPSE (http://glimmpse.samplesizeshop.org), our free and web application that calculates power and sample size for study designs with normally distributed outcomes. GLIMMPSE provides researchers with a user-friendly interface for calculating power and sample for the general linear multivariate model and the general linear mixed model with and without a Gaussian covariate. A full description of GLIMMPSE appears in [3].

GLIMMPSE Lite is a prototype mobile application that implements a subset of the functionality of GLIMMPSE. Limiting GLIMMPSE Lite to ANOVA designs enabled us to solve the general design challenges of porting statistical power software to mobile platforms with minimum development time. Using the experience we gained from developing GLIMMPSE Lite, we plan to expand the functionality of the mobile application to include multilevel and longitudinal designs in future releases.

### Organization of the manuscript

The remainder of the manuscript is organized as follows. The next section presents a review of statistical power software packages available for standard web browsers, personal computers, and mobile platforms. In the Challenges section, we describe general solutions to the computing challenges listed above, and then the specific solutions we used to implement GLIMMPSE Lite. Finally, in the Conclusions section we present conclusions and discuss directions for further research in this area.

## Review of power and sample size software

### Statistical power and sample size software packages

The credibility of a statistical software package primarily depends on three factors: 1) if the user can clearly understand what statistical methods were chosen to perform the power and sample size calculations, 2) if the user can understand what computational technologies were used, and 3) if experiments were used to validate the power and sample size results. SPSS [4], Power and Precision [5], NQuery [6], PASS [7], SAS PROC GLMPOWER [8], POWERLIB [10], Optimal Design [11], UnifyPow [12], and GLIMMPSE [3] all fit the criteria for credibility.

Statistical power software packages that are distributed as stand-alone desktop installations can cost upwards of $500, even for an academic version. These packages include SPSS [4], Power and Precision [5], NQuery [6], PASS [7], and SASGLMpower [8]. Several statistical power software packages are available for free, including POWERLIB [10], Optimal Design [11], UnifyPow [12], and GLIMMPSE [3]. Although these packages are available for free, some require an active license for an underlying statistical software package. For example, the POWERLIB software requires a paid installation of SAS/IML [9].

Usability differs across these software packages. Specifically, POWERLIB requires knowledge of SAS/IML programming statements. UnifyPow must be installed on the user's workstation, has no graphical user interface, and was last updated in 2008. GLIMMPSE, our free, web-based application, can be accessed via a standard web browser and is easy to use with no prior hands-on training.

Several other statistical power software packages that are available for free are listed in [13]–[15]. Many of these packages provide none or very limited validation information.

### Statistical power applications for mobile platforms

As part of our design process, we surveyed all statistical packages for mobile devices available on the App Store [16] or Google Play [17] store as of November 1, 2012. **Table 1** provides a review of the applications, including information about the mobile platform supported, the cost, and the capability of each application. Our audit of competing software shows that no other mobile application performs power and sample size calculations for ANOVA. In addition, most mobile statistical software is relatively expensive.

**Table 1:** Summary of statistical applications available on Apple iOS and Google Android mobile platforms.

Product Name	Platform	Cost	1-sample t-test	2-sample t-test	1-way ANOVA	Data Analysis	Power Analysis	Sample Size Analysis
GlimmpseLite	iOS/Android	FREE	-	✓	✓	-	✓	✓
ANOVA	Android	$2.99	-	-	-	-	-	-
1-way ANOVA	iOS	$8.99	-	-	✓	✓	-	-
ANOVA	iOS	$4.99	-	✓	✓	✓	-	-
ANOVA	Android	$2.99	-	✓	✓	✓	-	-
Power Analysis	iOS	$4.99	✓	✓	-	-	✓	✓

## Challenges in migrating software to a mobile platform

### Introduction

As outlined in the Introduction section, migrating a statistical power software package to a mobile platform raises several challenges. In this section, we describe each challenge, discuss possible solutions, and then present the specific solutions we adopted to develop GLIMMPSE Lite.

### C1. Statistical power software is computationally intensive, and many mobile devices have limited computing resources

Power and sample size calculations require computationally intensive algorithms including matrix multiplication, matrix inversion, numerical integration, and search. To give an idea of the difficulty of the problem, the complexity of matrix multiplication is 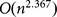 for Coppersmith-Winograd [18], where *n* is the largest dimension of the matrices. Thus, calculating power and sample size becomes increasingly slow as the size of the experiment increases, even on a powerful desktop machine. [3] provided timing data for sixteen example power and sample size analyses (http://samplesizeshop.org/documentation/glimmpse/validation-results/). Total CPU time to calculate power using GLIMMPSE was as much as 101 seconds per calculation for complex designs. The timing experiments were performed on an Intel i7-2600 quad core, 3.40 GHz processor with 8GB of RAM, running 64-bit Windows 7.

By contrast, a typical fast mobile platform comes equipped with 1GB of RAM and a processor speed of 1100 MHz. The lower RAM and slower processors on a mobile device would lead to unacceptably slow calculation times, if the calculation were performed on the mobile device. There are two approaches to solving the computational speed issue. One approach is to invest strong efforts to optimize the algorithm. In the case of power calculations, this is impossible, as the inherent complexity is irreducible. The other option is to export the calculation to another more powerful machine, as is done in the client-server model. The mobile device accesses a web service to perform the power and sample size calculations. Because the web server has fewer limitations in RAM and processing power, the approach is faster. The mobile device needs only to elicit inputs to the power calculation from the user, format the request to the server, and display the results.

**Figure 1:**
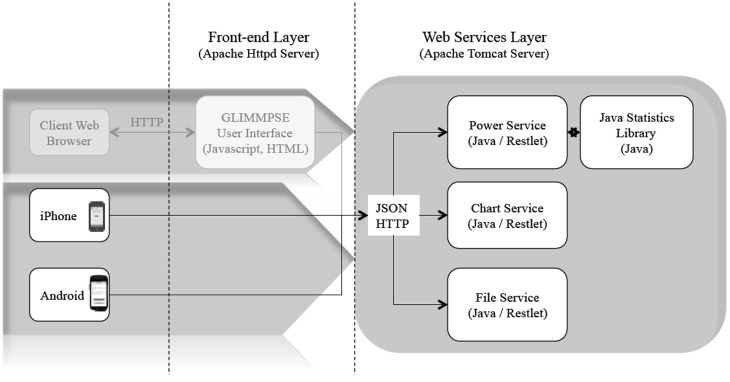
Current web and mobile software architecture.

When designing GLIMMPSE Lite, we decided to use a client-server approach based on the Java web services architecture [19]. This approach afforded several advantages. We minimized slow calculations on the mobile platform by shifting complex calculations to the server. We were able to leverage the existing, validated, web services architecture developed for our web-based product, GLIMMPSE. We reduced our development time significantly through extensive code reuse. **Fig. 1** presents the software architecture used by both GLIMMPSE Lite and GLIMMPSE.

The architecture contains three web services. The *Power Service* calculates power and sample size. The *Chart Service* produces power curves. The *File Service* provides upload and save functionality. For full details on the GLIMMPSE software architecture, see http://samplesizeshop.com/documentation/glimmpse/. GLIMMPSE Lite currently interacts only with the *Power Service*. Thus, the ANOVA power and sample size calculations are performed on the server side, rather than overburdening the mobile device.

### C2. Statistical power software requires complex inputs

Statistical power software involves a complex set of inputs. The user must choose the Type I error rate, list the dependent and independent variables, and specify hypotheses, means, variances, relative group sizes, correlations, repeated measures, covariates, and the type of statistical test. To assist users in creating a study design, a simple and easy to understand user interface was needed. To address the challenge, we took the following approach: 1) We divided the input requests so that the user only had to understand and input one item on each screen; 2) We added logic checks to prevent the user from entering nonsensical input; and 3) We tested the resulting user interface in a focus group.

We implemented GLIMMPSE Lite on two smartphone mobile platforms: iOS and Android. Our choice of these two platforms is based on their current share of the worldwide mobile phone market (see C6 under the Challenges section for details). Creating a usable interface required different design decisions for each platform. To illustrate the design decisions for each platform, we describe how to specify an ANOVA study design using GLIMMPSE Lite on each mobile platform.

**Figure 2:**
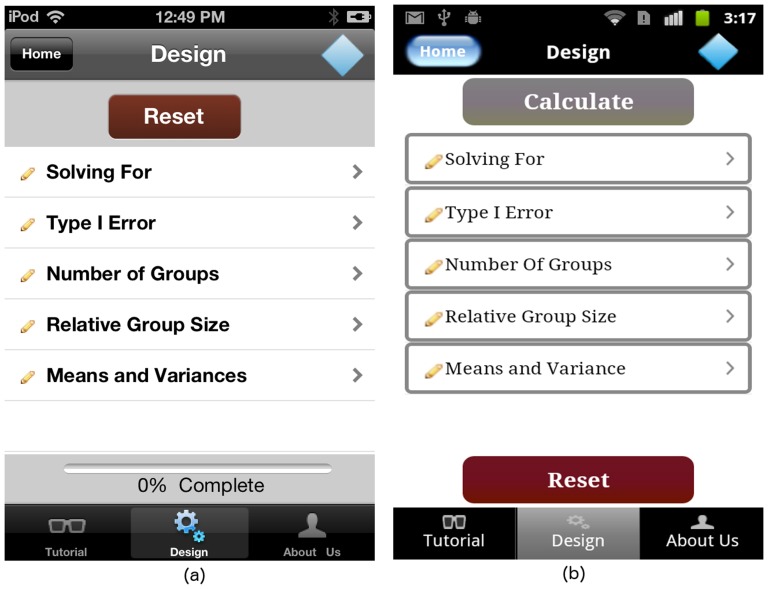
ANOVA “Design” screen on (a) iOS and (b) Android platforms.

**Figure 3:**
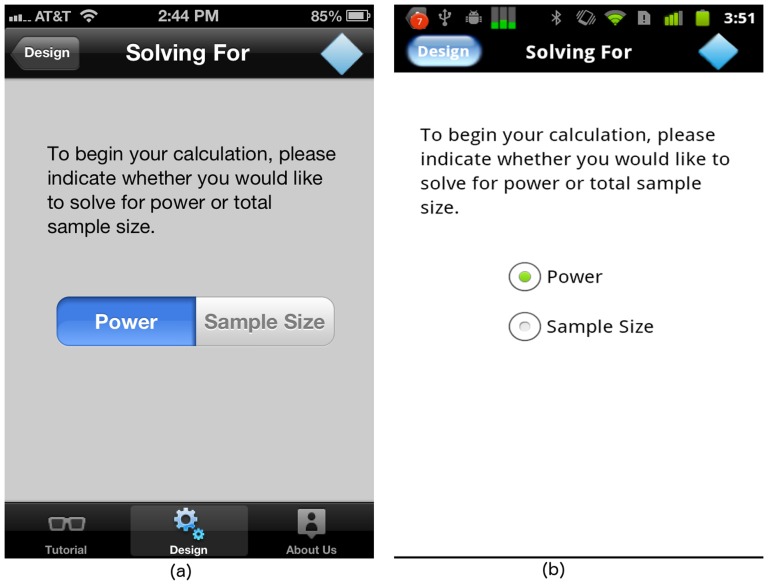
“Solving For” input screen on (a) iOS and (b) Android platforms.

The GLIMMPSE Lite user interface first presents the “Design” screen, a top-level list of the required inputs for an ANOVA design, in a tabular format, as shown in **Fig. 2**. Each line of the table shown in **Fig. 2** loads a new screen, which allows users to input a specific design feature. Users can navigate easily back and forth from any input screen to the “Design” screen, as shown in **Fig. 2**. For example, a tap on the table cell titled “Solving For” in **Fig. 2** loads the screen shown in **Fig. 3**. The “Design” button on the upper left of the “Solving For” screen provides navigation back to the “Design” screen.

#### Setting up an ANOVA design on GLIMMPSE Lite

While the GLIMMPSE Lite user interface is similar for both platforms (iOS and Android), a few design solutions are specific to the SDK available for each platform, as discussed below.

#### The “Solving For” screen

To start the ANOVA study design, the user first selects whether to solve for power or sample size. To facilitate the selection, we use the “Segmented Control” widget from the iOS SDK, as shown in **Fig. 3(a)**. Since the same widget is not available on the Android platform, we use radio buttons instead, as shown in **Fig. 3(b)**.

**Figure 4:**
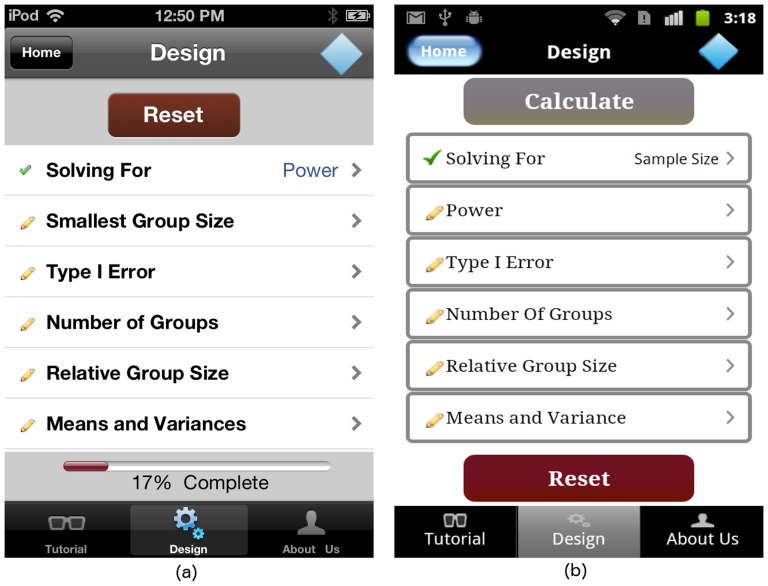
“Design” screen tailored to the user's choice of “Solving For” on (a) iOS and (b) Android platforms.

Each time the user selects power or sample size, the “Design” screen in **Fig. 2** is updated to reflect the selection made by user, as shown in **Fig. 4**. If solving for sample size, the user is required to specify the desired power level. When solving for power, the user must input the smallest group size. The “Design” screen summarizes the study design, providing a visual indicator for input completion. Specifially, the pencil image for each list entry on the “Design” screen changes to a green checkmark, indicating that the corresponding portion of the study design is complete.

#### The “Type I Error” screen

**Figure 5:**
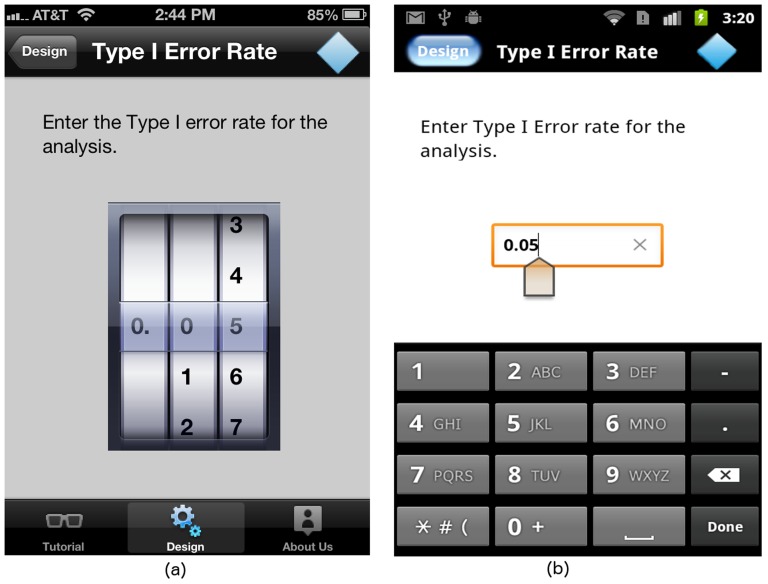
“Type I error” screen on (a) iOS and (b) Android platforms.

The Type I error rate is the probability of rejecting a null hypothesis when in fact the null hypothesis is true. The Type I error rate is a number between 0 and 1. For the iPhone, we use the “Rotating Wheel” widget available on iOS, shown in **Fig. 5(a)**. For the Android platform, we use a text box instead, as shown in **Fig. 5(b)**.

#### The “Number of Groups” screen

**Figure 6:**
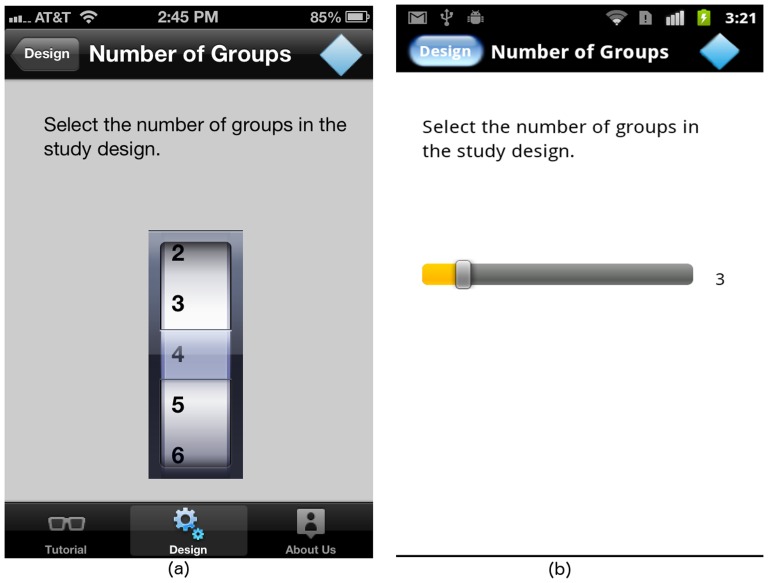
“Number of Groups” screen on (a) iOS and (b) Android platforms.

A one-way ANOVA design compares means among two or more groups. Currently, GLIMMPSE Lite supports up to 10 groups. As shown in **Fig. 6**, we use the iPhone “Rotating Wheel” widget, and a text box for the Android to enable the user to select the number of groups involved in the study design.

#### The “Relative Group Size” screen

**Figure 7:**
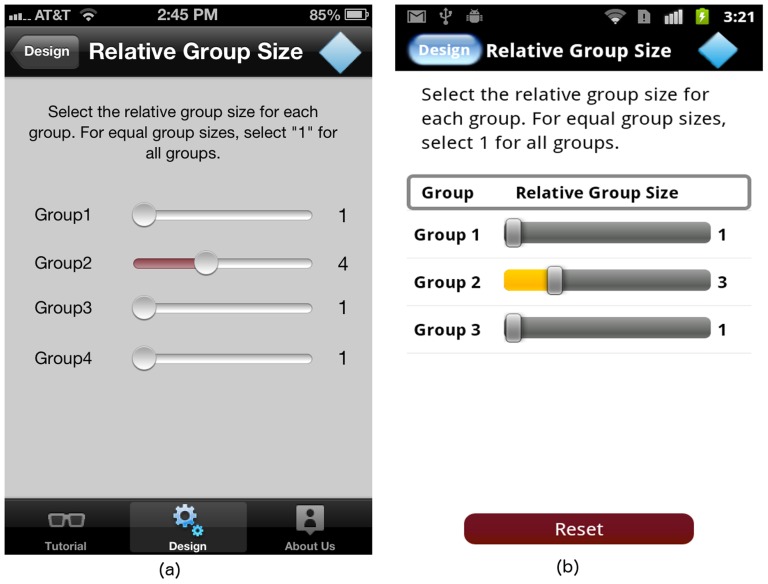
“Relative Group Size” screen on (a) iOS and (b) Android platforms.

GLIMMPSE Lite supports power and sample size for ANOVA designs with equal or unequal group sizes. The default study design assumes equal group sizes. For unequal group sizes, GLIMMPSE Lite sliding bars allow the user to select the relative size of the groups (see **Fig. 7**). Users can slide a bar from left to right to increase the relative group size.

#### The “Smallest Group Size” screen

**Figure 8:**
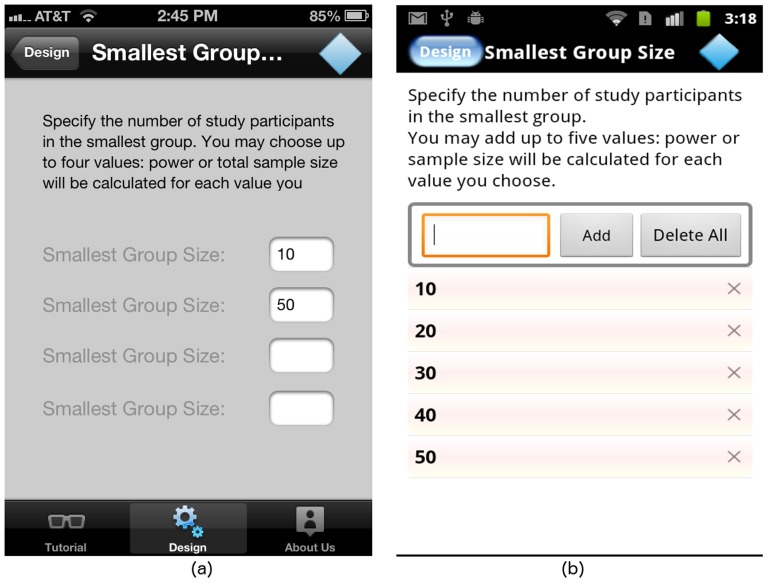
“Smallest Group Size” screen on (a) iOS and (b) Android platforms.

If solving for power, the user must specify the smallest group size. Currently, GLIMMPSE Lite allows users to enter up to 4 smallest group sizes. A separate power calculation is performed for each group size. Since a user may enter any integer value for this input, we provide text boxes on both iOS and Android platforms, as shown in **Fig. 8**.

#### The “Power” screen

**Figure 9:**
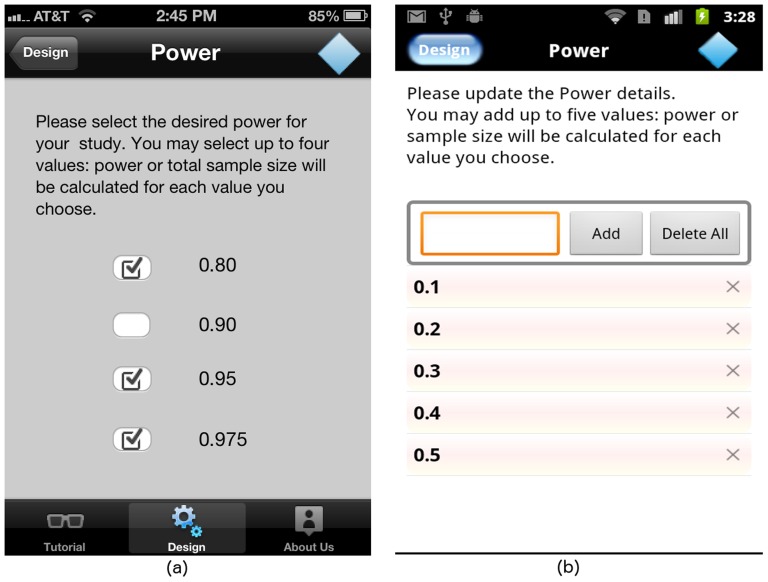
“Power” screen, if solving for sample size on (a) iOS and (b) Android platforms.

If solving for sample size, the user must input the desired power level for the ANOVA study design. Currently, GLIMMPSE Lite allows users to select up to four commonly used power levels on each platform, as shown in **Fig. 9(a)** for the iOS platform, and in **Fig. 9(b)** for the Android platform. A sample size is calculated for each power value provided.

#### The “Means and Variances” screen

**Figure 10:**
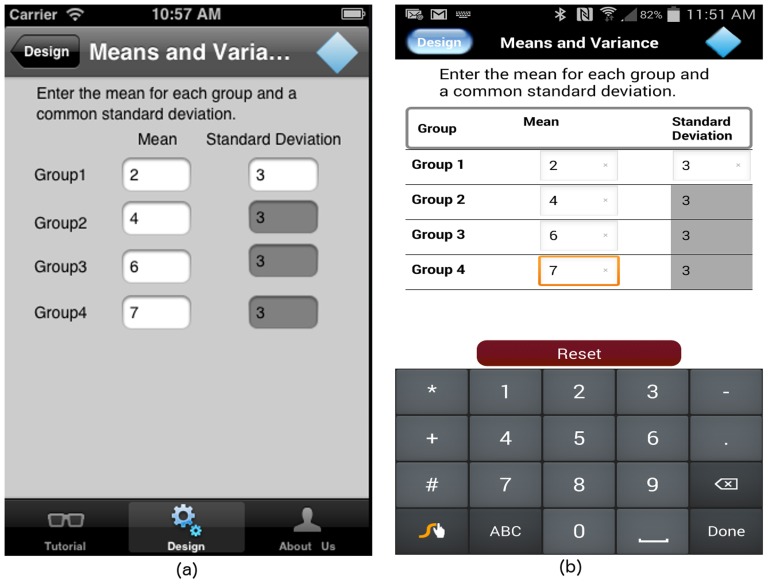
“Means and Variances” screen on (a) iOS and (b) Android platforms.

GLIMMPSE Lite assumes equal variance across all groups. The user enters a mean value for each group and a single standard deviation value. Since the user can enter any real value for means and any positive number for the standard deviation, we use text boxes on both iOS and Android platforms, as shown in **Fig. 10**.

**Figure 11:**
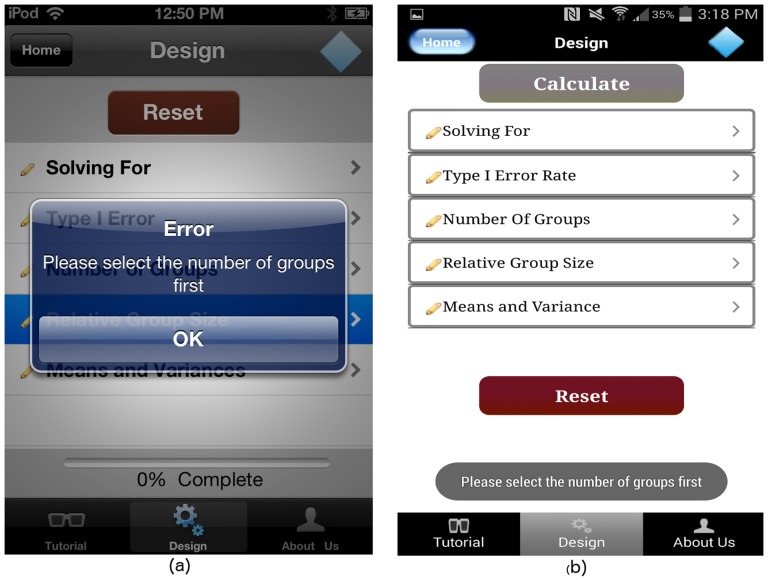
Example error dialog on (a) iOS and (b) Android platforms.

**Figure 12:**
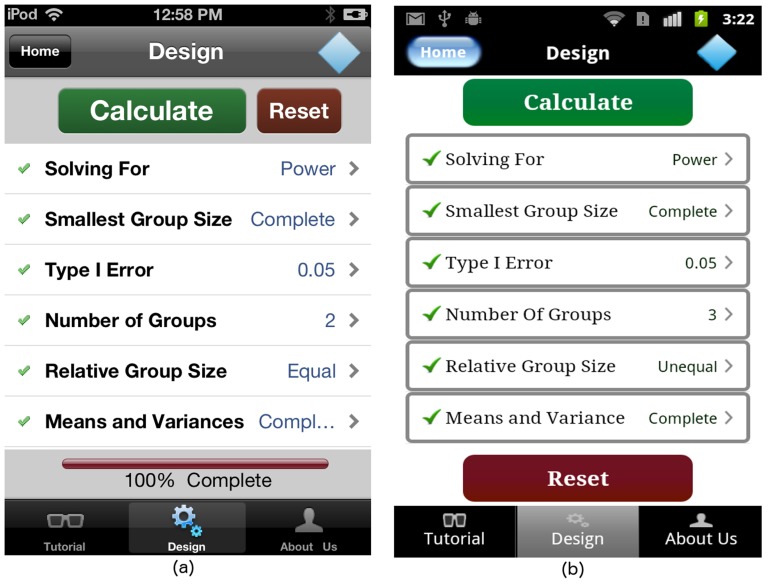
“Design” screen with completed ANOVA study design on (a) iOS and (b) Android platforms.

The GLIMMPSE Lite user interface allows the user to select the “Relative Group Size” and “Means and Variances” screens after the “Number of Groups” input is complete. If a user taps on the cell titled “Relative Group Size” or “Means and Variances” without completing the “Number of Groups” input, the GLIMMPSE Lite interface alerts the user with an error message shown in **Fig. 11**. Once all of the required inputs for the ANOVA study design are complete, a “Calculate” button appears on the “Design” screen as shown in **Fig. 12**.

#### Focus group study

To evaluate the GLIMMPSE Lite user interface, we conducted a focus group study with nine participants. To match our target market, the focus group included five PhD level biostatisticians, three PhD level epidemiologists, and a Master's level student in biostatistics. The goal of the focus group was to assess the usability, look and feel, and text readability of the GLIMMPSE Lite user interface. Participants used a Likert scale [20] to rate agreement with the following statements about the iOS and Android versions of GLIMMPSE Lite:

- The look and feel of the GLIMMPSE Lite application was appealing.
- I could easily read the text even on the small screen.
- My calculation was successful.

**Figure 13:**
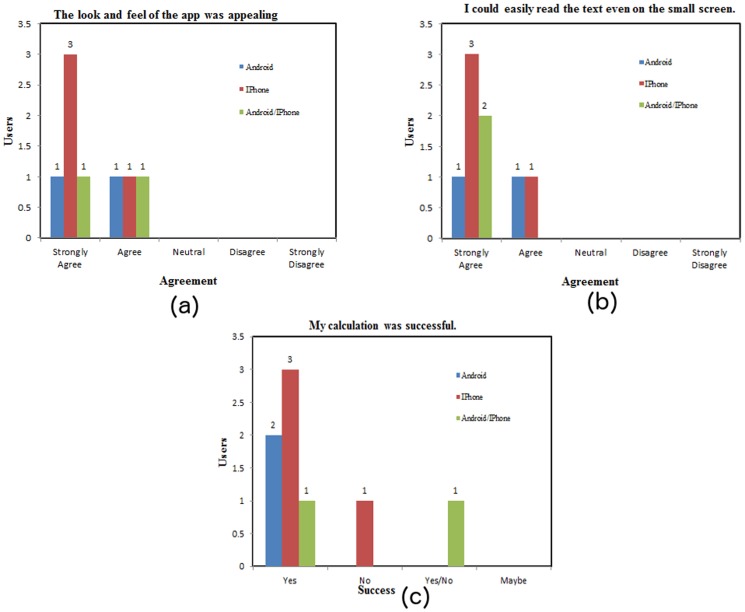
Focus group results for GLIMMPSE Lite on iOS and Android platforms.

**Fig. 13** presents the results of our focus group analysis. The user satisfaction was high for both “look and feel” and “text readability” for both mobile platforms. Two users encountered errors while using the application, which we have since corrected.

### C3. The interface must provide an appropriate level of guidance to users regarding the required inputs

Many users of our software have limited experience with power or sample size calculations. They may not be trained in statistical concepts such as the Type I error rate or power. Adding help pages is the obvious solution. However, on a mobile platform, the amount of help is limited by the mobile device screen size.

To address this challenge, we implemented a separate help page for each input screen. Separating the help pages from the input screens allows the GLIMMPSE Lite user interface to access information only when needed, while maintaining a clean design. The help page is available for all users upon request. The text was designed to be understandable for individuals with limited statistical training, while still providing adequate theoretical details for trained scientists and statisticians. Sophisticated or experienced users who need no help can proceed directly to the input screen without looking at the help pages.

**Figure 14:**
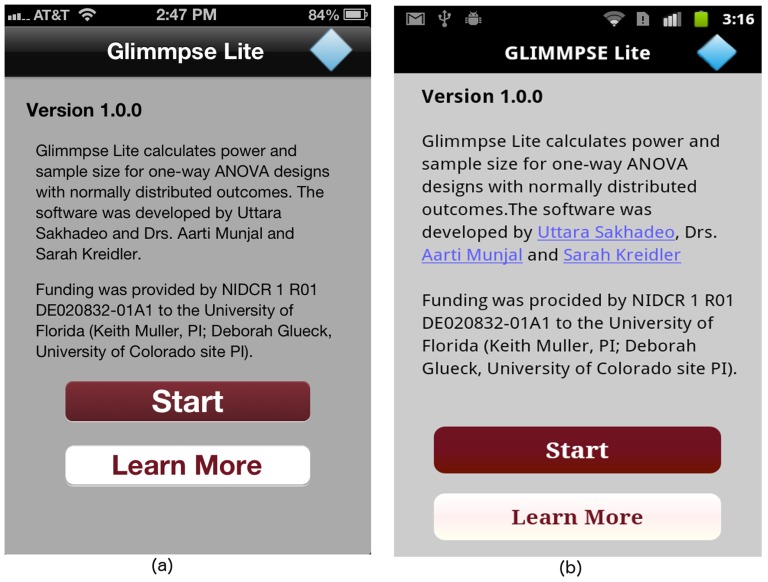
“Start” screen on (a) iOS and (b) Android platforms.

**Figure 15:**
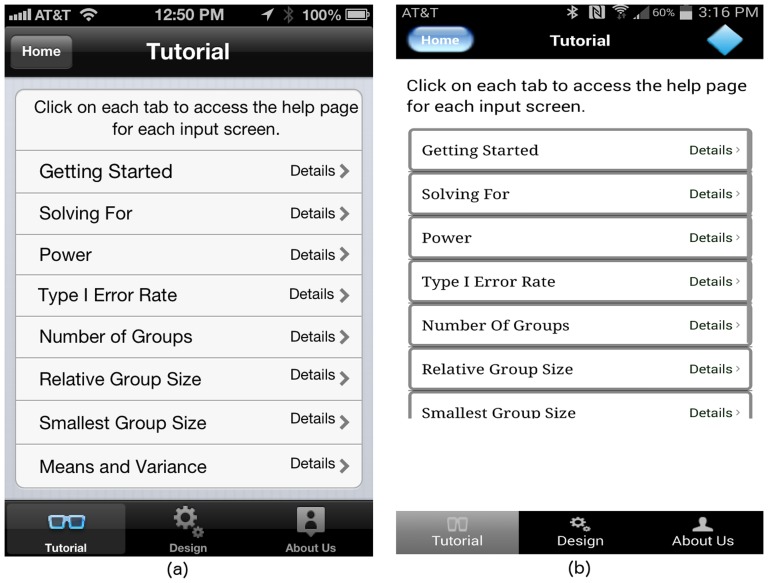
“Tutorial” screen on (a) iOS and (b) Android platforms.

Users can navigate to the help pages in one of two ways. They can go directly to the help page from the first screen. They can also view the help screens from anywhere within the input sequence, without losing any study design inputs. The start screen of the GLIMMPSE Lite user interface is shown in **Fig. 14**. The “Start” button navigates directly to the “Design” screen, thereby allowing frequent GLIMMPSE Lite users to begin their ANOVA study design without pausing to look at a tutorial. The “Learn More” button, on the other hand, brings the user to the “Tutorial” screen, as shown in **Fig. 15**. The “Tutorial” screen is more appropriate for first-time GLIMMPSE Lite users, as it provides generic information related to the ANOVA study design inputs.

**Figure 16:**
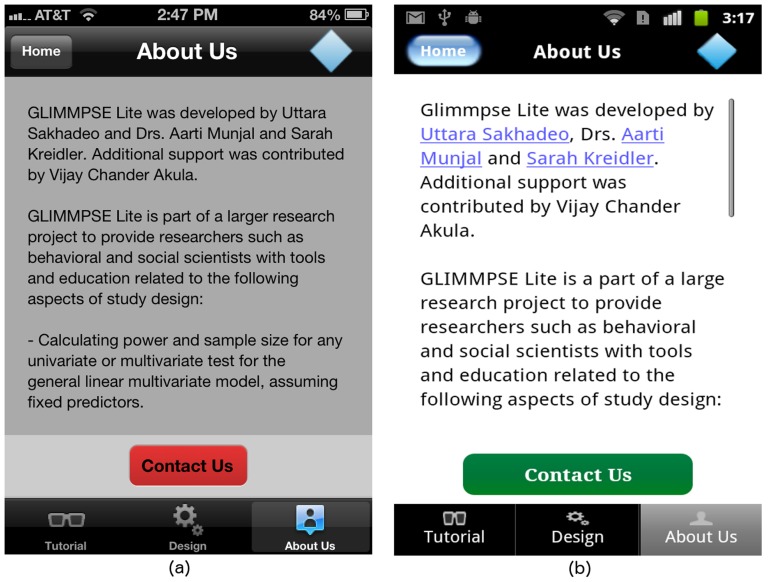
“About Us” screen on (a) iOS and (b) Android platforms.

Users who need assistance during the input sequence can always access the help pages by tapping one of three tabs displayed at the bottom of the screen as shown in **Fig. 15**. The purpose of each tab is described below:

1. The **Tutorial** tab leads to a table showing each available help page.
2. The **Design** tab summarizes the study design created so far, lists finished inputs, and indicates which screens remain to be filled in.
3. The **About Us** tab includes funding information and details on our development team. GLIMMPSE Lite users can submit a query or report a bug via the “Contact Us” button provided at the bottom of the “About Us” screen (see **Fig. 16**).

### C4. Communication between the mobile device and supporting servers must be fast and lightweight

The client-server model used in GLIMMPSE Lite is only beneficial if the mobile application can communicate efficiently with the server. HTTP [21] is a reasonable protocol to use for communication since most mobile devices are already web-enabled. The primary design decision for the communication layer that remained was the choice of the textual data format. The ideal format must be lightweight, supported on multiple platforms, human readable, and easy to parse. Possible data formats include direct Java object serialization [22], XML [23], and JSON [24]. Java object serialization is an extremely lightweight binary format, but is not human-readable. In addition, object serialization may lead to platform compatibility problems. XML is robust, human readable, and cross-platform. However, it can be overly verbose, and parsing is expensive. On the other hand, JSON provides a lightweight communication medium and maintains human readability. JSON parsing toolkits are available for most mobile platforms.

**Figure 17:**
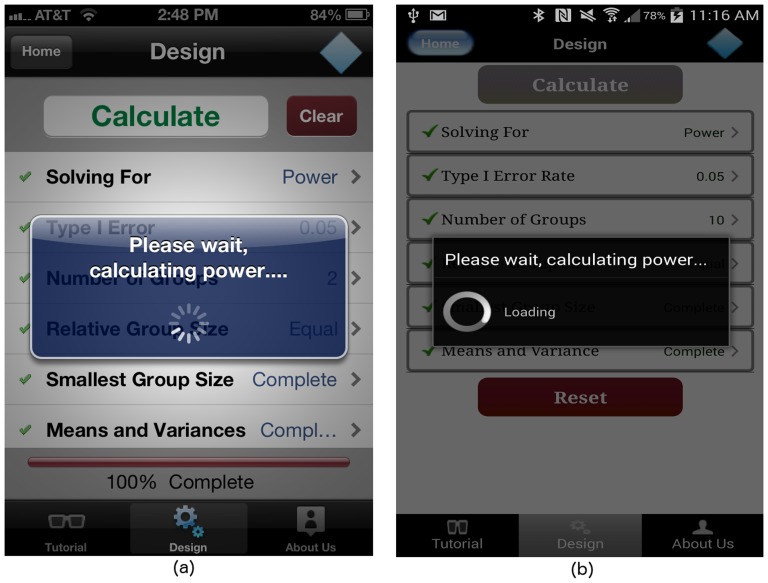
Processing dialog box on (a) iOS and (b) Android platforms.

**Figure 18:**
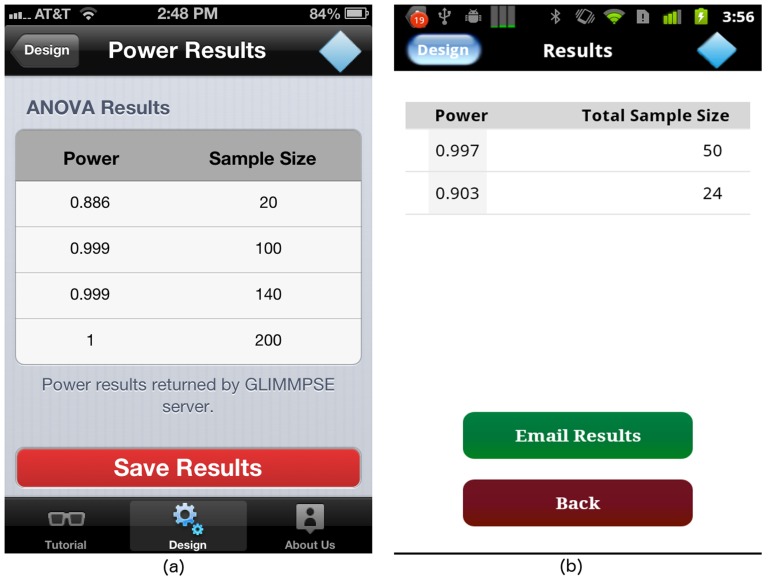
“Results” screen on (a) iOS and (b) Android platforms.

For GLIMMPSE Lite, we selected JSON as the textual data format. When the user has finished describing the ANOVA study design, the “Calculate” button at the top of the “Design” screen is highlighted as shown in **Fig. 12**. When the user taps on the “Calculate” button, GLIMMPSE Lite creates a study design object, encodes the object into JSON, and sends an HTTP request to the *Power Service*, shown in **Fig. 17**. When the calculation is complete, the *Power Service* sends a JSON encoded list of results back to the mobile device. After receiving the server's response, the GLIMMPSE Lite user interface extracts the power and sample size values from the JSON encoded object and displays these as a two-column table on the “Results” screen, as shown in **Fig. 18**.

Platform specific libraries for iOS and Android support JSON encoding and HTTP requests. For the iOS platform, we use a third-party framework called AFNetworking [25] to communicate with the *Power Service*. The AFNetworking framework supports JSON encoding and, thus, enables the GLIMMPSE Lite user interface to serialize the study design object before sending a HTTP request to the web server. For Android, JSON encoding and HTTP requests are supported by the Restlet library [22].

### C5. The mobile app must scale easily and robustly

**Figure 19:**
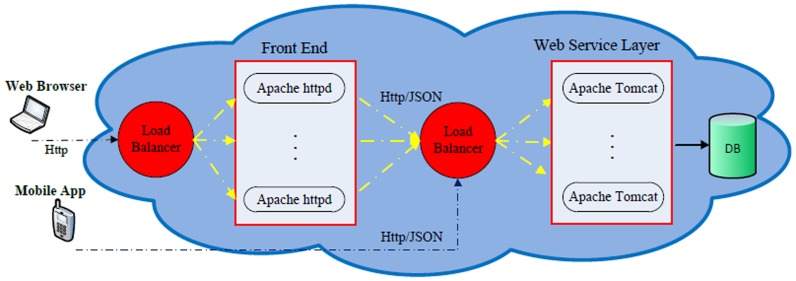
Our scaled web and mobile software architecture.

GLIMMPSE Lite relies on our web services architecture, shown in **Fig. 19**. As more and more users hit the mobile site, calculation times may slow, and the system may crash. We fear that frequent crashes due to traffic or server load inevitably will erode the user base for the statistical application.

To address the challenge of scalability, our web services are hosted on a reliable cloud-based architecture provided by Amazon Web Services (AWS) [26], as shown in **Fig. 19**. The cloud-based service provided by Amazon offers several infrastructural as well as architectural advantages. AWS is cost-effective, easily customized, and can be quickly scaled to handle increased user demand.

### C6. Supporting multiple platforms requires significant development time

As discussed in the Introduction section, each mobile platform has its own software development kit. In other words, native applications developed for one mobile platform will not compile on other mobile platforms. Therefore, the development time for a mobile application increases proportionally to the number of supported mobile platforms.

**Figure 20:**
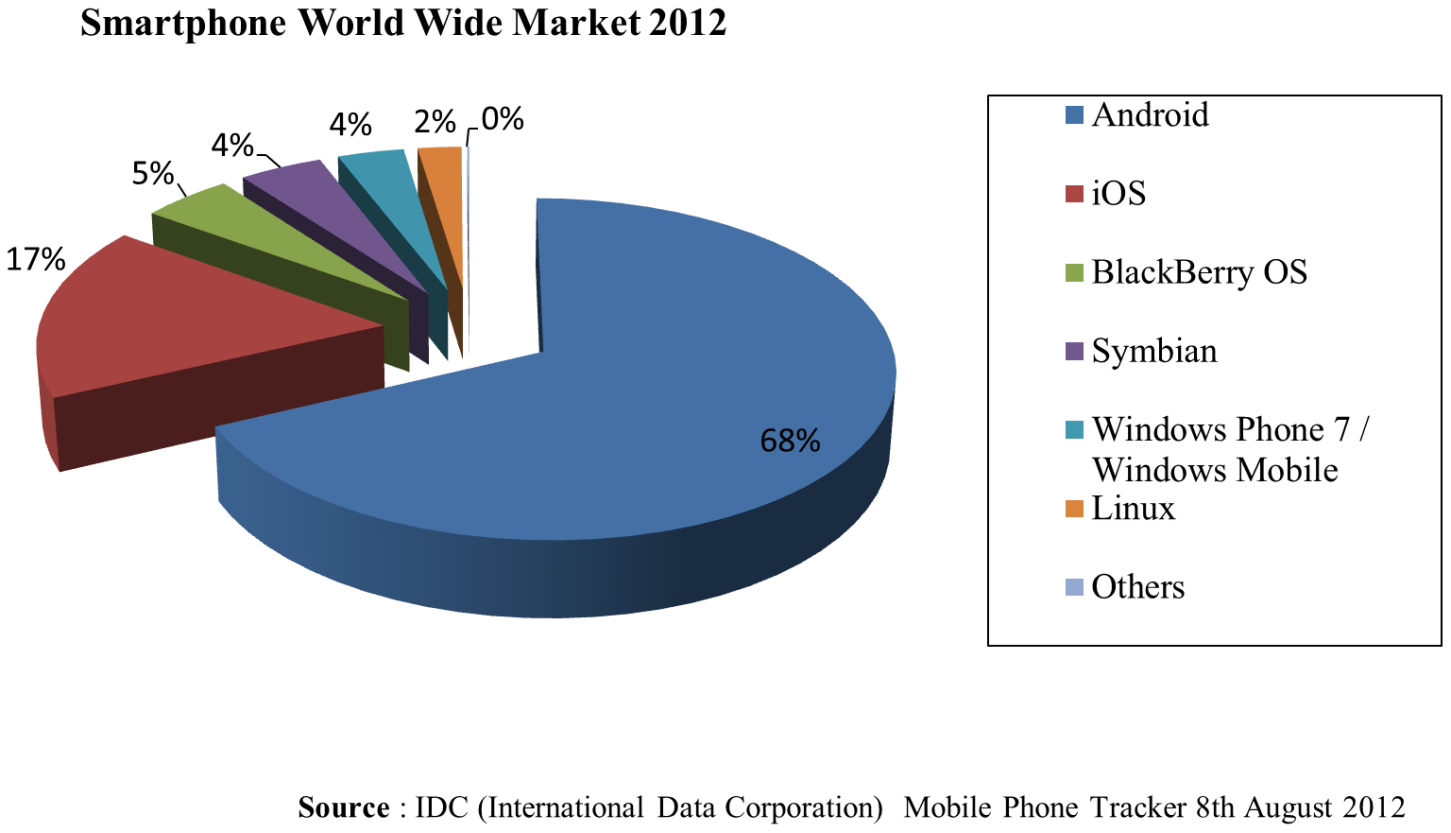
Smartphone World wide market share 2012.

With limited resources on our development team, we chose to develop GLIMMPSE Lite only for the iOS and Android mobile platforms. **Tables 2** and **3** list the tools used in developing GLIMMPSE Lite for each platform. These two platforms together hold 85% of the world-wide mobile market, as shown in **Fig. 20**. GLIMMPSE Lite was developed by our two part-time software engineers in 2 months, and required roughly 720 woman-hours for the entire development process, from design to final release. GLIMMPSE Lite is built as a native application on both iOS and Android mobile platforms. Thus, GLIMMPSE Lite required separate code branches for the iOS platform using Objective-C, Xcode [27], and AFNetworking, and for the Android platform using Java, Eclipse [28], and Restlet.

**Table 2:** Development environment for the iOS platform.

Term	Definition
.app	The application file created for release.
iOS	The iPhone Operating System.
Xcode IDE	Integrated development environment for iOS devices.
iOS 5 - iOS 6	The supported Apple mobile device operating system versions.
Objective-C	The programming language used for native app development.
Provisioning profile	The developer profile for build, test, and release on multiple iOS devices.
Certificate	The developer certificate purchased for development from Apple.

**Table 3:** Development environment for the Android platform.

Term	Definition
.apk	The application package file created for release.
Dalvik	The Android platform's virtual machine (VM).
Eclipse IDE	The Indigo Release 1
adb	Android debug bridge, a command line debugging application.
Android SDK	The Android software development kit.
API Level	An integer value that defines the framework API revision offered.
minSDKVersion (9)	The minimum SDK version supported by the application.
targetSDKVersion (15)	The target SDK version for the application designed to run.
AVD	The Android virtual devices.

The GLIMMPSE Lite application for both iOS and Android platforms is available at http://www.samplesizeshop.org/. The Android version can be installed directly from Google Play. The iOS version is available from the App Store.

### C7. Mobile devices have frequent updates in operating systems

Mobile devices have a rapid development cycle, and mobile operating system change frequently. New operating systems may be unstable, and manufacturers may not fix problems on a regular schedule. Therefore, computer scientists must decide what subset of mobile platforms they can afford to support. The problem is further complicated by the fact different variants of the operating system may only run on specific phone hardware. Even after the target operating systems and devices are chosen, developers must test the mobile application on all combinations of the supported operating systems, mobile devices, and software development kits.

We felt that supporting iOS and Android would cover the largest number of mobile users while not overburdening the development team. We have released our GLIMMPSE Lite application for iOS versions 5 and 6 for iPhone and iPod touch devices. iOS 5 is considered a stable iOS release by Apple. The iPhone 5, the latest mobile phone released by Apple, runs iOS 6. We have tested our GLIMMPSE Lite application on both iOS 5 and 6 versions via two iPhone simulators (5.0 and 6.0), four physical iPhones, and two iPod touch devices.

The GLIMMPSE Lite application for the Android platform has been released for application programming interface (API) levels 9 (i.e., Gingerbread) onwards. We have tested our GLIMMPSE Lite application via Android Virtual Devices for Gingerbread with API levels 9 and 10 [29], Honeycomb with API levels 11, 12 and 13 [30], Ice Cream Sandwich with API levels 14 and 15 [31], and Jelly Bean with API level 16 and 17 [32]. We have also tested the application on three physical smartphones, including the Google Samsung Nexus S (Gingerbread API level 10) [33], the Samsung S III (Jelly Bean API level 16) [34] and the Samsung Stratosphere (Gingerbread API level 9) [35].

### C8. Applications must interact effectively with other applications

Resources on a mobile device, such as random access memory (RAM), file storage, and internet bandwidth, are typically shared among multiple running applications. Therefore, a statistical power application must efficiently use the shared resources and gracefully handle errors caused by interaction with other applications installed on the mobile device.

The GLIMMPSE Lite product includes extensive error checking when interacting with other applications. For example, GLIMMPSE Lite allows users to save power results either by sending an email or by producing an image in the camera folder on the device. When a valid email cannot be found, GLIMMPSE Lite will alert the user with an appropriate error message, similar to that shown in **Fig. 11**, encouraging the user to set up an email client. Similarly, GLIMMPSE Lite will alert the user if the camera folder or other local storage is inaccessible. Lastly, to respect the user's privacy concerns, GLIMMPSE Lite always asks for the user's permission before accessing any other resources on the mobile device.

### C9. Users must be able to save outputs for later reference

Upon completion of a power or sample size calculation, GLIMMPSE Lite allows the user to save the results via the following methods:

#### Email Results

**Figure 21:**
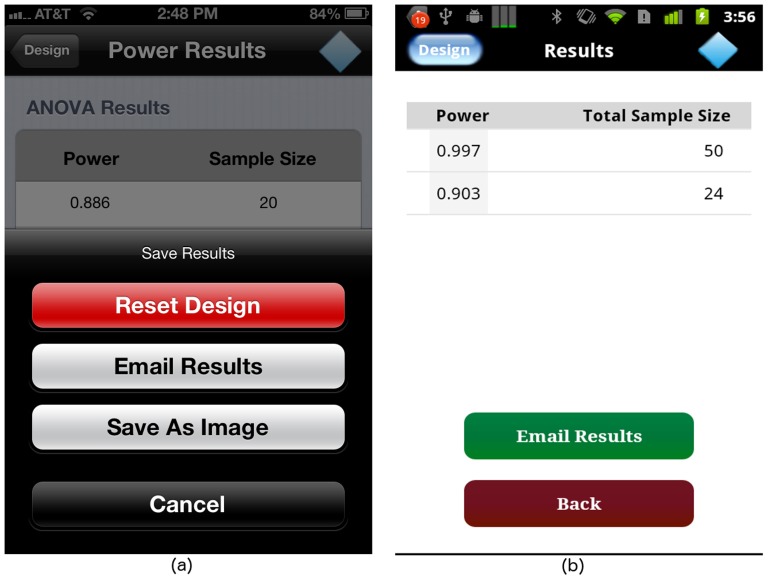
Save buttons on (a) iOS and (b) Android platforms.

Many users will want the ability to re-create a power analysis, as well as looking at the results of the power analysis in the future. We allow both activities. In case an internet connection is available (wi-fi or 3G/4G), the power and sample size results can be emailed via the default email client registered on user's mobile device, as shown in Fig. 21. The results email includes a.csv file with power and sample size results, as well as the subset of the input parameters. To re-run a power analysis, the user can request the JSON encoded study design object file (.json). The.json file can be uploaded at our main website, glimmpse.Samplesizeshop.org. Using the uploaded.json file, the user can recreate the power analysis exactly, or make small changes and run it again.

#### Save As Image

In case the mobile device loses an internet connection, the calculated power and sample size results can still be saved locally on the mobile device via the “Save As Image” option, as shown in Fig. 21a. This feature accesses the camera folder of the mobile device and saves a snapshot of the “Results” screen for later use.

### C10. Users must be able to reproduce the power and sample size results without reiterating the whole process

For credible studies, scientists and statisticians need to be able to save study designs and reproduce the power and sample size results. The GLIMMPSE software allows users to save their study design anytime during the design input process. The saved study design can be used later to reproduce the power and sample size results without following all the steps involved in the input process. The GLIMMPSE Lite user interface saves the ANOVA study design as a JSON file that can be used on the GLIMMPSE web application via the “Upload a study design” feature.

## Conclusions and future work

This paper described the challenges that occur when porting statistical power software to a mobile platform. We suggested specific solutions for each challenge, and gave examples from the development of GLIMMPSE Lite, our mobile ANOVA power and sample size application.

Our next effort in this direction is to implement GLIMMPSE Mobile. GLIMMPSE Mobile will allow users to calculate power and sample size for multilevel and longitudinal studies. Currently, GLIMMPSE Lite is available on only iOS and Android mobile platforms. To increase the user base for GLIMMPSE Mobile and make power and sample size calculations more accessible to users, we will target the development of GLIMMPSE Mobile to multiple mobile platforms. For this purpose, we will use Phonegap [36], an open-source framework capable of building cross platform mobile applications. Phonegap allows the development of mobile applications in HTML [21], Java, and CSS [37]. The same code can be rebuilt for multiple mobile platforms, including iOS, Android, Blackberry OS [38], Windows Phone [39], Palm webOS [40], Bada [41], and Symbian [42].

While GLIMMPSE Lite does not currently provide power curves, the underlying architecture will allow us to easily implement these capabilities for GLIMMPSE Mobile in the future. If a future GLIMMPSE Mobile release requests a power curve, the existing *Chart Service* can generate a power curve image in response to an HTTP GET request.

With the release of GLIMMPSE Lite, we have provided a free, open-source, validated power and sample size tool for users seeking to design ANOVA studies. In addition to being a useful tool for scientists, we hope that the explanations of our design decisions provide a guide for other designers seeking to produce statistical software for mobile platforms.
